# Translation and validation of the Dari version of the hospital survey on patient safety culture for healthcare settings in Afghanistan

**DOI:** 10.1038/s41598-025-13461-x

**Published:** 2025-07-26

**Authors:** Javad Moghri, Jamshid Jamali, Laleh Satarzadeh

**Affiliations:** 1 Social Determinants of Health Research Center, Mashhad University of Medical Sciences, Mashhad, Iran; 2 Department of Health Economics and Management Sciences, School of Health, Mashhad University of Medical Sciences, Mashhad, Iran; 3 Department of Biostatistics, School of Health, Mashhad University of Medical Sciences, Mashhad, Iran; 4 Student Research Committee, Mashhad University of Medical Sciences, Mashhad, Iran; 5 Medical Faculty, Ghalib University, Herat, Afghanistan

**Keywords:** Patient safety, Translation, Validation, Psychometrics, Afghanistan, Health care, Health occupations, Medical research, Risk factors

## Abstract

Patient safety culture (PSC) is a critical determinant of healthcare quality, as it influences error reduction and clinical outcomes. The hospital survey on patient safety culture (HSOPSC), a globally validated tool for assessing PSC, was translated and validated in Afghanistan a setting facing unique healthcare challenges, including resource constraints and a fragmented healthcare system. The translation adhered to WHO guidelines, involving forward–backward translation, expert review, and pilot testing with nurses and midwives at one of Afghanistan’s largest hospitals, located in the capital city. Psychometric evaluations included face, content, and construct validity. Reliability was assessed through internal consistency (Cronbach’s α, Omega coefficients) and test–retest reliability. The 42-item questionnaire demonstrated robust validity and reliability. Content Validity Index (CVI) scores ranged from 0.82 to 1.0, exceeding acceptable thresholds, while the Content Validity Ratio (CVR) ranged from 0.64 to 1.0, meeting required standards. Face validity was confirmed by an impact score > 1.5 (range 1.86–3.96). Two items from the Staffing dimension exhibited factor loadings below the recommended threshold of 0.4 and were removed, leading to the exclusion of this dimension (fewer than three retained items). Overall reliability was strong, with Cronbach’s α = 0.848 and Omega = 0.841. The validated Dari version of the HSOPSC comprises 11 dimensions after removing Staffing. It demonstrates good validity and reliability, establishing its suitability for patient safety research in Afghanistan.

## Introduction

Patient safety is a fundamental component of high-quality healthcare systems, playing a crucial role in minimizing preventable harm and improving clinical outcomes^1^. The increasing complexity of medical interventions, coupled with rapid technological advancements, has heightened the need for robust patient safety culture (PSC) within healthcare organizations^2^. A strong PSC—defined as shared values, attitudes, and practices that prioritize safety—has been linked to reduced medical errors, enhanced staff communication, and improved patient outcomes^3^. Conversely, weak safety cultures contribute to adverse events, leading to increased morbidity, mortality, and financial burdens on healthcare systems^4^.

To assess PSC, various measurement tools have been developed, with the Hospital Survey on Patient Safety Culture (HSOPSC) being one of the most widely used instruments globally. Studies in multiple countries, including Iran^5^, China^6^, and Saudi Arabia^7^, have validated its reliability and applicability in evaluating safety culture dimensions such as teamwork, error reporting, and organizational learning.

The inclusion of 12 dimensions in HSOPSC is grounded in a comprehensive understanding of healthcare system complexities and the multifaceted nature of safety culture^8^. These dimensions, developed and refined by the Agency for Healthcare Research and Quality (AHRQ), capture essential organizational, unit-level, and interpersonal factors empirically linked to patient safety outcomes^8^. The rationale for their inclusion stems from extensive psychometric testing, theoretical frameworks of organizational safety, and cross-cultural validation studies, ensuring the instrument measures critical aspects of safety culture relevant across diverse healthcare settings globally.

Teamwork within units (F1) and Teamwork across units (F9) are foundational dimensions, as effective collaboration is a well-established predictor of error prevention and response. Research consistently identifies teamwork as one of the strongest dimensions in safety culture surveys globally, reflecting its universal importance in coordinating care and mitigating risks associated with handoffs or communication breakdowns between departments^9,10^. Conversely, Handoffs and transitions (F11) is frequently identified as a weakness across studies^10,11^, highlighting systemic vulnerabilities in information transfer during critical points like shift changes or patient transfers. Including this dimension allows organizations to target specific communication processes for improvement, as poor handoffs are a known contributor to adverse events. Communication openness (F7) and Feedback and communication about error (F6) address the flow of safety-related information. Openness ensures staff feel safe to speak up about concerns, while feedback ensures that reported errors lead to visible changes, reinforcing a learning culture rather than a blaming one. Studies, including those in Peru and Estonia, demonstrate that these dimensions are crucial for psychological safety and continuous learning, though their measurement can be sensitive to phrasing, especially for negatively worded items requiring careful cultural adaptation during translation^12,13^.

Non-punitive response to error (F12) is critical for fostering psychological safety and encouraging error reporting. This dimension consistently scores among the lowest globally^9,10^, indicating a pervasive challenge where staff fear blame, leading to underreporting of incidents and missed learning opportunities. Its inclusion directly targets a cultural barrier to transparency. Organizational learning–continuous improvement (F3) represents the organizational capacity to learn from mistakes and implement changes. This dimension is typically a strength^9,10^, reflecting healthcare’s inherent focus on improvement, and serves as a key indicator of a proactive safety culture. Management support for patient safety (F4) and Supervisor/manager expectations and actions promoting safety (F2) measure leadership commitment at different levels. Senior management support (F4) provides resources and strategic direction, while direct supervisor actions (F2) translate policies into daily practices. Research shows that visible leadership commitment correlates with stronger overall safety perceptions and staff compliance with safety protocols^11,14^.

Staffing (F10) addresses workload adequacy, a structural factor impacting safety. Chronic understaffing is a global concern and consistently emerges as a critical weakness in safety culture surveys, including in Pakistan and Ghana^9,11^. Overworked staff are more prone to errors and less able to engage in safety practices, making this dimension vital for identifying resource-related risks. Overall perceptions of patient safety (F5) provides a summative evaluation of safety effectiveness within the unit or organization, reflecting the cumulative impact of other dimensions. It acts as a key outcome measure linked to actual safety performance data^9,14^. Finally, Frequency of events reported (F8) serves as a behavioral indicator of the safety culture’s effectiveness. Higher reporting rates suggest trust in non-punitive systems and engagement in safety improvement, providing a tangible metric to assess the impact of interventions aimed at improving dimensions like non-punitiveness and organizational learning^13,14^.

The selection and retention of these twelve dimensions are further justified by robust psychometric testing. Confirmatory Factor Analysis (CFA) in large-scale validations, such as the Swedish study involving over 84,000 respondents, generally supports the factor structure, though some adaptations (like adding dimensions for local legal requirements) occur while maintaining the core framework^14^. Cross-cultural validation studies, from Malaysia to Estonia, demonstrate that while minor linguistic or item-level adjustments are often needed, these core dimensions remain conceptually relevant and psychometrically sound across diverse healthcare systems and languages^12,13,15^. The dimensions allow for benchmarking within and between organizations and tracking progress over time, fulfilling core purposes of safety culture assessment: diagnosis, intervention targeting, and evaluation. Therefore, the twelve dimensions of the HSOPSC collectively provide a validated, comprehensive, and actionable framework for understanding and improving the complex socio-technical system that underpins patient safety.

In Afghanistan, where the healthcare system faces significant challenges-including resource limitations, workforce shortages, and infrastructural constraints-assessing PSC is critical for identifying systemic gaps and implementing targeted improvements^16^. However, research on PSC in Afghanistan remains scarce, and no validated Dari-language tool exists to facilitate such assessments. Given linguistic and cultural differences, direct translation of instruments without proper validation may compromise their reliability and relevance^17^.

This study aims to translate and validate the Dari version of the HSOPSC, ensuring its cultural adaptation and linguistic appropriateness for Afghanistan’s healthcare context. By developing a psychometrically sound tool, this research will enable policymakers and healthcare administrators to evaluate PSC effectively, identify areas for improvement, and ultimately enhance the quality of care. The findings will contribute to the limited body of literature on patient safety in conflict-affected settings and support evidence-based interventions to strengthen Afghanistan’s healthcare system.

## Materials and methods

### Study population

This cross-sectional study was conducted in 2020 at Istiqlal Hospital, the largest public hospital in Kabul, the capital of Afghanistan. Dari, also known as Dari Persian or Eastern Persian, is one of Afghanistan’s two official languages, the other being Pashto. While often referred to as “Afghan Persian” by Western reports, the Dari language is known as Dari by the official government. It is a lingua franca, or common language of communication between ethnic groups in Afghanistan, and is a close relation to Tajik Persian. A fact of significance to the initiate is that Dari is the same as Persian spoken in Iran but with variations in regional vocabulary and pronunciation^18–20^. Participants included nurses and midwives actively employed at the hospital. Ethical approval was obtained from the Ethics Committee of Mashhad University of Medical Sciences, Iran (IR.MUMS.FHMPM.REC.1403.119), and informed consent was secured from all participants.

### Translation process

The Hospital Survey on Patient Safety Culture (HSOPSC) was translated into Dari following World Health Organization (WHO) guidelines for cross-cultural adaptation. The process included:

1. Forward Translation: Two independent native Dari-speaking translators, fluent in English, generated initial translations. Discrepancies were resolved through consensus to produce a unified Dari version.
2. Backward Translation: Two bilingual translators, blinded to the original English version, independently back-translated the Dari draft into English.
3. Expert Panel Review: A panel comprising translators, authors, and two patient safety experts compared all versions (original, forward, backward) to resolve semantic discrepancies and finalize a pre-test draft.
4. Pilot Testing: Ten nurses and midwives completed the pre-test draft. Ambiguous terms were revised based on feedback.
5. Final Backward Translation: An independent bilingual translator back-translated the finalized Dari version into English to confirm conceptual equivalence.

### Validity assessment

#### Face validity

Face validity is the degree to which questionnaire items superficially appear to reasonably measure the construct of interest, in the opinion of respondents and/or experts. It is a subjective judgment determining the relevance, clarity, and appropriateness of the items in the target population and setting. Face validity simply means whether the questionnaire “looks like” it is measuring what it should measure, as a first check before more rigorous validation procedures are conducted^21,22^. In this study, face validity was assessed using both quantitative and qualitative approaches.

##### Quantitative

Thirty nurses and midwives rated the importance of each item on a 5-point Likert scale. An impact score > 1.5 (calculated as: [frequency of “important” ratings × mean importance]/total participants) confirmed item clarity and relevance^23^.

##### Qualitative

Patient safety experts assessed phrasing and cultural appropriateness of terms.

#### Content validity

Content validity pertains to the extent to which an instrument comprehensively includes all the essential elements necessary to accurately assess the targeted construct or domain. This assessment considers practical aspects such as relevance and necessity, ensuring that the instrument provides a complete and appropriate measurement of the specific aspect of cognition or impairment within a given context. From an expert’s perspective, content validity guarantees that all relevant facets are adequately represented and detailed, minimizing the risk of omitting significant components of the construct being measure^22,24^. Eleven specialists (≥ 2 years of hospital experience, familiarity with HSOPSC) rated item relevance and necessity. Content Validity Ratio (CVR) and Content Validity Index (CVI) were calculated using Lawshe’s table (CVR ≥ 0.59 for 11 experts) and a 4-point scale (CVI ≥ 0.79 acceptable)^25^.

#### Construct validity

Construct validity in a questionnaire is the extent to which a questionnaire accurately measures what theory or construct it is purported to measure. It measures whether the items within the questionnaire actually address the underlying abstract theme or concept that the researcher is trying to address. In short, it’s about ensuring the questionnaire measures what it’s supposed to measure and not something else^22,26^. Confirmatory factor analysis (CFA) was performed on the responses of 267 participants from the HSOPSC questionnaire, which was distributed to 300 individuals, resulting in a response rate of 89%. Model fit was assessed using indices (1 < χ^2^/df < 3, CFI, TLI > 0.90; RMSEA < 0.08). Factor loadings < 0.4 prompted item removal. The HOELTER test was employed to evaluate the adequacy of the sample size in the context of CFA^27^.

### Reliability assessment

#### Internal consistency

Internal consistency of a questionnaire may be defined as the extent to which all of the items or questions representing the same construct provide the same result. It measures the homogeneity of a multi-item scale and the extent to which the different components of the questionnaire are measuring the same construct. Essentially, it is checking whether all of the questions are “pulling in the same direction” and measuring the same thing^22,28^. Cronbach’s α and McDonald’s Omega (ω) were calculated. Values > 0.7 indicated acceptable reliability. Omega was prioritized for its robustness to scale dimensionality^29^.

#### Test–retest reliability

Test–retest reliability is a statistical measure used to evaluate the stability and consistency of a measurement over time. It assesses the degree of agreement between results obtained from the same participants on two separate occasions, typically spaced two weeks apart, assuming that the conditions remain unchanged. High test–retest reliability indicates that the instrument yields stable and reproducible results across different time points^22,30^. Thirty participants completed the questionnaire twice over a 2-week interval. Intraclass Correlation Coefficient (ICC; two-way mixed model, absolute agreement) evaluated stability (ICC > 0.75 acceptable)^30^.

### Data analysis

Analyses were conducted in SPSS (v26) and AMOS (v24). Descriptive statistics summarized participant characteristics. CFA evaluated dimensionality, while reliability coefficients (α, ω, ICC) quantified consistency.

## Results

### Translation and cultural adaptation

The HSOPSC was successfully translated into Dari using the World Health Organization (WHO) guidelines^31^. All items demonstrated linguistic appropriateness and cultural relevance, with no significant discrepancies identified during forward–backward translation or expert panel review. The Dari version of the HSOPSC questionnaire is provided in the Supplementary Appendix.

### Face validity

Face validity was evaluated quantitatively through an impact score (IS) calculated from ratings by 30 nurses and midwives. The mean age was 36.2 ± 10.2 years, and the mean years of work experience were 9.9 ± 8.4 years. Female participants comprised 43.3% (13 individuals), and 83.3% (25 individuals) were married. Regarding educational background, 36.7% (11 participants) held a Bachelor’s degree, 13.3% (4 participants) had a license, 30.0% (9 participants) possessed an MD degree, 3.3% (1 participant) held a master’s degree, and 16.7% (5 participants) had specialized training or a PhD. All items achieved IS > 1.5 (range 1.86–3.96), indicating clarity and perceived importance. Qualitative feedback from patient safety experts further confirmed the cultural and linguistic appropriateness of terms (Table 1).

**Table 1:** Face, Content, and Construct validity indices results.

F	Item	Impact score	CVR	CVI (relevancy)	Initial loading factor	Final loading factor*	Result
F1 Teamwork	F1_1	2.10	0.82	0.91	0.814	0.805	Confirmed
	F1_2	1.86	0.82	0.91	0.754	0.744	Confirmed
	F1_3	2.52	0.82	0.82	0.802	0.833	Confirmed
	F1_4	3.87	1.00	1.00	0.741	0.745	Confirmed
F2 Supervisor expectations and actions	F2_1	2.66	0.64	1.00	0.881	0.916	Confirmed
	F2_2	3.28	1.00	0.91	0.708	0.714	Confirmed
	F2_3	3.44	1.00	0.82	0.770	0.792	Confirmed
	F2_4	3.28	0.82	0.82	0.871	0.881	Confirmed
F3 Organizational learning	F3_1	2.80	0.82	0.82	0.750	0.740	Confirmed
	F3_2	3.36	1.00	0.82	0.734	0.758	Confirmed
	F3_3	2.10	0.64	0.91	0.702	0.735	Confirmed
F4 Management support	F4_1	2.59	0.82	1.00	0.733	0.743	Confirmed
	F4_2	2.59	0.82	1.00	0.822	0.848	Confirmed
	F4_3	2.73	0.64	0.82	0.707	0.742	Confirmed
F5 Overall perceptions	F5_1	2.73	0.82	0.91	0.795	0.824	Confirmed
	F5_2	2.52	0.82	0.82	0.703	0.731	Confirmed
	F5_3	2.73	0.64	0.91	0.714	0.753	Confirmed
	F5_4	3.12	0.64	0.91	0.791	0.787	Confirmed
F6 Feedback and communication	F6_1	2.80	0.82	1.00	0.863	0.874	Confirmed
	F6_2	2.80	1.00	1.00	0.879	0.906	Confirmed
	F6_3	3.12	0.64	1.00	0.803	0.841	Confirmed
F7 Communication openness	F7_1	2.87	0.82	0.82	0.704	0.733	Confirmed
	F7_2	2.66	0.64	0.91	0.779	0.802	Confirmed
	F7_3	1.98	0.64	1.00	0.864	0.865	Confirmed
F8 Frequency of reported	F8_1	2.52	0.64	0.82	0.726	0.725	Confirmed
	F8_2	2.04	0.82	1.00	0.899	0.938	Confirmed
	F8_3	2.22	0.82	0.82	0.894	0.906	Confirmed
F9 Teamwork across units	F9_1	3.20	0.64	0.91	0.797	0.828	Confirmed
	F9_2	2.52	0.82	1.00	0.851	0.873	Confirmed
	F9_3	2.80	0.82	1.00	0.757	0.790	Confirmed
	F9_4	3.28	0.82	0.82	0.844	0.858	Confirmed
F10 Staffing	F10_1	2.28	1.00	1.00	0.708	–	Confirmed
	F10_2	1.86	0.64	0.82	**0.326**		Not confirmed
	F10_3	1.98	0.64	0.82	**0.259**		Not confirmed
	F10_4	2.59	0.82	0.91	0.726		Confirmed
F11 Handoffs and transitions	F11_1	3.87	1.00	1.00	0.893	0.894	Confirmed
	F11_2	3.12	0.82	0.91	0.881	0.908	Confirmed
	F11_3	3.28	0.82	1.00	0.826	0.818	Confirmed
	F11_4	3.36	0.64	1.00	0.729	0.754	Confirmed
F12 Non-punitive response	F12_1	3.96	0.64	1.00	0.707	0.704	Confirmed
	F12_2	2.59	0.82	0.91	0.785	0.793	Confirmed
	F12_3	3.36	0.64	1.00	0.742	0.757	Confirmed

Items with factor loadings below the acceptable threshold of 0.4 are highlighted.

*Standardized factor loading values after removing the Staffing dimension from the HSOPSC questionnaire.

### Content validity

Content validity was assessed via the Content Validity Ratio (CVR) and Content Validity Index (CVI). All 42 items met or exceeded the minimum thresholds (CVR ≥ 0.59 for 11 experts; CVI ≥ 0.79). The mean age of the experts participating in the content validity assessment was 45.13 ± 12.34 years. Four participants (36.4%) were female. Regarding their educational qualifications, two (18.2%) held a master’s degree, while the remaining experts held a PhD. The average scores across items were 0.79 (CVR) and 0.92 (CVI), confirming strong relevance and necessity (Table 1).

### Construct validity

This phase involved 267 participants whose mean age and work experience were 36.50 ± 10.71 and 9.32 ± 9.14 years, respectively. Of the participants, 52.4% (140 individuals) were female and 71.9% (194 individuals) were married. In terms of educational qualifications, 41.2% (110 participants) possessed an associate’s degree, while 10.5% (28 participants) held a bachelor’s degree. Additionally, 30% (80 individuals) of the participants were general practitioners, whereas 14.6% (39 individuals) were specialist physicians.

Two items in the Staffing dimension exhibited factor loadings < 0.4 (Item 2: 0.326; Item 3: 0.259). Following Kenny’s recommendation for a minimum of three items per dimension, the Staffing dimension was excluded, resulting in an 11-dimension structure (Table 1).

The goodness of fit indices for the CFA after remove staffing dimension indicated an acceptable model fit, with a chi-square to degrees of freedom ratio (χ^2^/df) of 2.812, a Comparative Fit Index (CFI) of 0.971, a Tucker-Lewis Index (TLI) of 0.959, and a Root Mean Square Error of Approximation (RMSEA) of 0.0052, all of which fell within acceptable limits. The HOELTER test identified the minimum adequate sample size for the CFA in this study as 254 participants at a 5% significance level, indicating that the sample size of 267 participants was sufficient for the analysis. The standardized factor loading values for the remaining dimensions are given in Table 1.

### Internal consistency

The overall internal consistency was strong (Cronbach’s α = 0.848; McDonald’s Omega = 0.841). All retained dimensions demonstrated α > 0.7, except Staffing (α = 0.62), reinforcing its exclusion (Table 2). Participants in the internal consistency assessment were identical to those involved in the face validity assessment.

**Table 2:** Reliability indices results.

Factor		α	ω
F1	Teamwork within units	0.703	0.607
F2	Supervisor/manager expectations and actions promoting patient safety	0.815	0.886
F3	Organisational learning—continuous improvement	0.704	0.737
F4	Management support for patient safety	0.818	0.851
F5	Overall perceptions of patient safety	0.735	0.682
F6	Feedback and communication about error	0.894	0.909
F7	Communication openness	0.875	0.857
F8	Frequency of events reported	0.777	0.839
F9	Teamwork across units	0.760	0.852
F10	Staffing	0.643	0.727
F11	Handoffs and transitions	0.783	0.735
F12	Non-punitive response to errors	0.708	0.659
Overall		0.848	0.841

Cronbach’s Alpha and omega values greater than 0.70 are considered as satisfactory, and 0.80 as excellent.

### Test–retest reliability

The Intraclass Correlation Coefficient (ICC) for the 30 participants assessed over a 2-week interval was 0.881 (95% CI 0.79–0.93), confirming excellent temporal stability. The same participants who took part in the face validity assessment also participated in the Test–Retest Reliability evaluation.

## Discussion

The Dari version of the Hospital Survey on Patient Safety Culture (HSOPSC) demonstrated robust psychometric properties, aligning with validations in Iran^5^, China^6^, and Saudi Arabia^7^. High reliability (Cronbach’s α/Omega > 0.8 for most dimensions) mirrored findings from the original AHRQ studies^8^, reinforcing its utility in diverse settings. Construct validity, assessed via confirmatory factor analysis (CFA), showed strong model fit (CFI = 0.95, RMSEA = 0.06), consistent with benchmarks for cross-cultural adaptations^32^ and comparable to Korean^33^ and Bulgarian^34^ versions.

The Staffing (F10) dimension from the HSOPSC comprises four items assessing workforce adequacy: Item F10_1 (“We have enough staff to handle the workload”), Item F10_2 (“Staff in this unit work longer hours than is best for patient care”), Item F10_3 (“We use more agency/temporary staff than is best for patient care”), and Item F10_4 (“We work in ‘crisis mode’ trying to do too much, too quickly”). In this study, Item F10_2 (factor loading: 0.326) and Item F10_3 (factor loading: 0.259) fell below the conventional 0.40 threshold for adequate construct representation^35^. This indicates these items failed to statistically cohere with the latent “Staffing” construct in this sample. Low loadings suggest contextual factors—such as normalization of extended hours or inconsistent use of agency staff—may have decoupled these items from respondents’ perception of safety-specific staffing challenges. Such psychometric instability aligns with cross-cultural validations; for example, Malaysian and Estonian HSOPSC adaptations similarly reported weak loadings for Item F10_3, with cross-loading onto resource management constructs^13,15^. Best practices in scale validation support excluding items with loadings < 0.40 to ensure discriminant validity^35^. Consequently, the Staffing dimension was excluded due to low validity (α = 0.64; factor loadings < 0.4), a challenge also observed in Croatia^36^ and Bulgaria^34^, where staffing metrics were culturally incongruent. While Staffing dimension exclusion is statistically justified, staffing’s substantive role in patient safety necessitates complementary assessment strategies. This exclusion likely reflects systemic issues in resource-limited, conflict-affected settings, such as chronic understaffing and burnout, which skew perceptions of workforce adequacy^16^. For instance, the Croatian HSOPSC reported α < 0.6 for both Staffing and Organizational Learning-Continuous Improvement (4), while the Korean version identified four dimensions (including Staffing) with reliability < 0.6^33^. These parallels underscore the need for context-specific adaptations in staffing-related metrics. Future refinements should explore items that capture workload-safety linkages without construct contamination.

The rigorous WHO-guided translation process addressed critical linguistic nuances, such as culturally resonant terms for “error reporting,” mitigating misinterpretation risks^17^. Items like “Staff fear blame for errors” highlighted Afghanistan’s punitive work culture a finding consistent with Iranian studies^5^, where blame-driven environments hindered error disclosure.

### Limitations and future directions

While this study provides the first validated PSC tool for Afghanistan, findings are constrained by single-site sampling (Istiqlal Hospital) and a cross-sectional design. Future work should expand to multi-center cohorts across public/private sectors to enhance generalizability. Longitudinal assessments are also needed to evaluate PSC evolution amid Afghanistan’s healthcare reforms.

### Policy and practical implications

The validated Dari HSOPSC equips policymakers to prioritize evidence-based interventions, such as nonpunitive error reporting and teamwork training dimensions with high reliability in this study. These align with WHO patient safety strategies and offer immediate pathways to strengthen PSC. The exclusion of the Staffing dimension further signals an urgent need for context-driven workforce metrics, a growing focus in global safety research.

## Conclusion

The Dari HSOPSC is a valid, reliable tool for assessing PSC in Afghanistan, though cultural adaptations for certain dimensions are essential. Findings mirror global trends while emphasizing context-specific challenges, offering a foundation to advance safety initiatives in conflict-affected health systems.

## Supplementary Information

Supplementary Information.

## Data Availability

The datasets generated and analyzed during this study are available from the corresponding author upon reasonable request, pending approval by the Mashhad Regional Committee for Medical Research Ethics.

## Abbreviations

PSC: Patient safety culture
HSOPSC: Hospital survey on patient safety culture
CVR: Content validity ratio
CVI: Content validity index
CFA: Confirmatory factor analysis
ICC: Intraclass correlation coefficient
