# Supplementary material for: Translation and validation of the Dari version of the hospital survey on patient safety culture for healthcare settings in Afghanistan
Source: Sci Rep. 2025 Jul 26;15:27277. doi: 10.1038/s41598-025-13461-x (PMC12297310; doi:10.1038/s41598-025-13461-x)
Supplement: Supplementary file 1 — Supplementary Information. [file 41598_2025_13461_MOESM1_ESM.pdf]

Appendix: English and Dari versions of HSOPSC questionnaire

**(Item) مورد**	**شماره (ID)**	**بعد (Dimension)**
English: People support one another in this unit.<br>دری: پرسونل این دیپارتمنت از یکدیگر حمایت می‌کُنند.	F1_1	F1
English: When a lot of work needs to be done quickly, we work together as a team to get the work done.<br>دری: وقتی ضرورت به اَنجام کارهای زیاد در مُدَتِ زمان کم باشَه، همه پرسنل منحیث یک تیم گرد هم می‌آیند تا کارها را با هم اَنجام دَهند.	F1_2	
English: In this unit, people treat each other with respect.<br>دری: در این بَخش پرسونل به یکدیگر با احترام برخورد می‌کُنند.	F1_3	
English: When one area in this unit gets really busy, others help out.<br>دری: وقتی یک قسمت تو این بَخش خیلی کار دارَد، بَخش‌های دیگر بهش کمک می‌کُنند.	F1_4	
English: My manager says a good word when he/she sees a job done according to established patient safety procedures.<br>دری: مدیر یا مسئول این بَخش، وقتی می‌بینَد که کاری مطابق با فرآیندهای مرتبط با مریض اَنجام شُدَه، از پرسونل تشکر می‌کُنَد و از آن‌ها تعریف یا ستایش می‌نماید.	F2_1	F2
English: My manager seriously considers staff suggestions for improving patient safety.<br>دری: مدیر یا مسئول این بَخش به جدیت به پیشنهاداتی که پرسونل برای بهبود مصونیت مریضان می‌دَهند توجه می‌کُنَد.	F2_2	
English: Whenever pressure builds up, my manager wants us to work faster, even if it means taking shortcuts.<br>دری: وقتی کار زیاد هست، مدیر یا مسئول بَخش می‌خواهد ما سریع‌تر کار کُنیم حتی اگه این به معنی انجام کار از راه‌های کوتاه یا میان بر باشد.	F2_3	
English: My manager overlooks patient safety problems that happen over and over.<br>دری: مدیر یا مسئول این بَخش، وقتی مُشکلات مرتبط با مصونیت مریض که بارها رخ می‌دَ، پیش می‌آد، چشم‌پوشی می‌کُنَد.	F2_4	
English: We are actively doing things to improve patient safety<br>دری: پرسونل در این بَخش به صورت فعال کارهای مختلف رو برای بهبود مصونیت مریض اَنجام می‌دَهند.	F3_1	F3
English: Mistakes have led to positive changes here.<br>دری: در این بَخش اشتباهات مُنجَر به ایجاد تغییرات مثبت می‌شَود.	F3_2	
English: After we make changes to improve patient safety, we evaluate their effectiveness.<br>دری: بعد از اُنکه بخاطر مصونیت مریض، تغییراتی ایجاد کَردیم، اثربَخشی تغییرات را مورد ارزیابی قرار می‌دهیم.	F3_3	
English: Hospital management provides a work climate that promotes patient safety.<br>دری: مدیریت شَفاخانه کاری را اَنجام میدهدَ که مصونیت مریض را بهبود ببَخشد.	F4_1	F4
English: The actions of hospital management show that patient safety is a top priority.<br>دری: اقدامات اداره شَفاخانه نشان میدهدَ که موضوع مصونیت مریض در شَفاخانه از اهمیت بسیار زیادی برخوردارَ و جزء اولویت‌های عمده شَفاخانه هست.	F4_2	
English: Hospital management seems interested in patient safety only after an adverse event happens.<br>دری: به نظر می‌رسَد که اداره شَفاخانه فقط بعد از اتفاق اُفتادن یه موضوع، به مسئله مصونیت مریض علاقه‌مند میشَه و توجه نِشُون میدَهد.	F4_3	
English: It is just by chance that more serious mistakes don’t happen around here.<br>دری: تنها چانس باعث می‌شَود که اشتباهات جدی‌تر در این بَخش اتفاق نَیافتد.	F5_1	F5
English: Patient safety is never sacrificed to get more work done<br>دری: مصونیت مریض هیچگاه بخاطر اَنجام کارهای بیشتر قربانی یا فراموش نمی‌شَود.	F5_2	
English: We have patient safety problems in this unit.<br>دری: در این بَخش مُشکلات مربوط به مصونیت مریض وجود دارَد.	F5_3	
English: Our procedures and systems are good at preventing errors from happening<br>دری: سیستم‌ها و پروسیجرهای موجود در این بَخش در جلوگیری از بروز خطاها خوب عَمل می‌کُنند.	F5_4	
English: We are given feedback about changes put into place based on event reports.<br>دری: در این بَخش، ما بر اساس گزارش‌های که دریافت می‌کُنیم، تغییراتی اِعمال می‌کُنیم و بازخورد به ما داده می‌شَود.	F6_1	F6
English: We are informed about errors that happen in this unit.<br>دری: ما از خطاها یا اشتباهاتی که در این بَخش اتفاق می‌افتَ خبردار می‌شویم.	F6_2	
English: In this unit, we discuss ways to prevent errors from happening again<br>دری: در این بَخش، ما در مورد راه‌های جلوگیری از تکرار خطاها یا اشتباهات صُحبَت می‌کُنیم.	F6_3	

English: Staff will freely speak up if they see something that may negatively affect patient care.<br>دری: پرسونل در صورت مشاهده کاری که ممکنَ برای مریض ایجاد مُشکل کُنَد، آزادانه، بدون تَرس و با صراحت سخن می‌گَویند.	F7_1	F7
English: Staff feel free to question the decisions or actions of those with more authority.<br>دری: پرسونل می‌تونَند بصورت آزادانه اقدامات و تصمیمات مسئولین شَفاخانه را مورد پرسش قرار بِدَهند.	F7_2	
English: Staff are afraid to ask questions when something does not seem right .<br>دری: پرسونل در مواردی که احساس می‌کُنن که بعضی کارها می‌تونَ مصونیت مریض را به مخاطره بی اَندازَ، از سوال کَردن و یا پُرسیدن دلیل آن هَراس دارَن.	F7_3	
English: When a mistake is made, but is caught and corrected before affecting the patient, how often is this reported?<br>دری: اگر یک اشتباه یا خطا پیش آید و قبل از اینکه مریض صدمه بِبینَد اصلاح شَه، خطا راپور داده میشَود؟	F8_1	F8
English: When a mistake is made, but has no potential to harm the patient, how often is this reported?<br>دری: هنگامیکه اشتباهی رخ می‌دَ، امّا آن اشتباه به اندازه ای مهم نیَست که به مریض صَدَمَه بِزَنه راپور داده می‌شَود.	F8_2	
English: When a mistake is made that could harm the patient, but does not, how often is this reported?<br>هنگامیکه اشتباهی رخ می‌دَهد که می‌توانَد مُنجَر به آسیب زَدن به مریض شَود، ولی در نهایت هیچ اتفاقی برای مریض نمی اُفتَد، راپور داده می‌شَود؟	F8_3	
English: Hospital units do not coordinate well with each other.<br>دری: بَخش‌های شَفاخانه با یکدیگر هماهنگی خوبی نَدارَند.	F9_1	F9
English: There is good cooperation among hospital units that need to work together.<br>دری: همکاری و تشریک مساعی خوبی بین بَخش‌های که ضَرورت به کار با یکدیگر دارَن وجود دارَد.	F9_2	
English: It is often unpleasant to work with staff from other hospital units.<br>دری: کار کردن با پرسونل بَخش های دیگر شَفاخانه برای ما ناخوشایندَ.	F9_3	
English: Hospital units work well together to provide the best care for patients.<br>دری: بَخش‌های شَفاخانه به منظور فراهم کردن بهترین مراقبت‌ها برای مریض به خوبی با یکدیگر کار می‌کُنند.	F9_4	
English: In this unit, we have enough staff to handle the workload.<br>دری: تعداد پرسونل کافی در این بَخش برای اَنجام کار هَست.	F10_1	F10
English: Staff in this unit work longer hours than is best for patient care.<br>دری: پرسونل تو این بَخش بیشتر از زمان دلخواه کار پرسونل برای مراقبت از مریض کار می‌کُنن.	F10_2	
English: We use more agency/temporary staff than is best for patient care<br>دری: ما از پرسونل موقت، بیشتر از اون چیزی که برای مراقبت دُرست از بیمار لازمه، استفاده می‌کُنیم.	F10_3	
English: We work in "crisis mode" trying to do too much, too quickly.<br>دری: ما در حالت بحران کار می کُنیم و تلاش می کُنیم تا کارهای زیادی را با سرعت بسیار زیاد اَنجام دَهیم.	F10_4	
English: Things “fall between the cracks” when transferring patients from one unit to another.<br>دری: در وقتی که بیمار از یک بَخش به بَخش دیگر مُنتقل می‌شَ، مُمکِنَ بعضی از چیزها رعایت نَشود.	F11_1	F11
English: Important patient care information is often lost during shift changes.<br>دری: معلومات مهم در مورد مراقبت مریض اغلبا در هنگام تبدیلی نوکریوالی به یکدیگر داده نمی‌شَود.	F11_2	
English: Problems often occur in the exchange of information across hospital units.<br>دری: بیشتر در تبادله‌ی اطلاعات بین بَخش‌های بیمارستان مُشکل پیش می‌آیَد.	F11_3	
English: Shift changes are problematic for patients in this hospital.<br>دری: تبدیلی نوکریوالان در این شَفاخانه برای مریضان ایجاد مُشکل می‌کُنَد.	F11_4	
English: Staff feel like their mistakes are held against them.<br>دری: پرسونل احساس می‌کُنن که اشتباهاتشون باعث می‌شَود، در موردشون به قِسمی یا نظری دیگر دیده شود.	F12_1	F12
English: When an event is reported, it feels like the person is being written up, not the problem.<br>دری: وقتی یک حادثه راپور داده میشود، احساس می‌شَود که بیشتر در مورد شخص تَذکر دادَ می‌شَود، نه در مورد مُشکل.	F12_2	
English: Staff worry that mistakes they make are kept in their personnel file.<br>دری: پرسونل نگرانِ این هَستَن که اشتباهاتشان در دوسیه سوانحشان دَرج گَردَ.	F12_3	
